# Understanding How the Subcommissural Organ and Other Periventricular Secretory Structures Contribute via the Cerebrospinal Fluid to Neurogenesis

**DOI:** 10.3389/fncel.2015.00480

**Published:** 2015-12-23

**Authors:** Maria M. Guerra, César González, Teresa Caprile, Maryoris Jara, Karin Vío, Rosa I. Muñoz, Sara Rodríguez, Esteban M. Rodríguez

**Affiliations:** ^1^Instituto de Anatomía, Histología y Patología, Facultad de Medicina, Universidad Austral de ChileValdivia, Chile; ^2^Departamento de Biología Celular, Facultad de Ciencias Biológicas, Universidad de ConcepciónConcepción, Chile

**Keywords:** cerebrospinal fluid, circumventricular organs, CSF-contacting neurons, subcommissural organ, SCO-spondin, transthyretin, integrins, neurogenesis

## Abstract

The dynamic and molecular composition of the cerebrospinal fluid (CSF) and, consequently, the CSF physiology is much more complex and fascinating than the simplistic view held for decades. Signal molecules either transported from blood to CSF or secreted into the CSF by circumventricular organs and CSF-contacting neurons, use the CSF to reach their targets in the brain, including the pre- and postnatal neurogenic niche. The subcommissural organ (SCO), a highly conserved brain gland present throughout the vertebrate phylum, is one of the sources for signals, as well as the choroid plexus, tanycytes and CSF-contacting neurons. The SCO secretes into the fetal and adult CSF SCO-spondin, transthyretin, and basic fibroblast growth factor. These proteins participate in certain aspects of neurogenesis, such as cell cycle of neural stem cells, neuronal differentiation, and axon pathfinding. Through the CSF, the SCO-secretory proteins may reach virtually any target in the embryonic and adult central nervous system. Since the SCO continues to secrete throughout life span, it seems likely that the neurogenetic property of the SCO compounds would be targeted to the niches where neurogenesis continues in adulthood. This review is aimed to bring into discussion early and new evidence concerning the role(s) of the SCO, and the probable mechanisms by which SCO compounds can readily reach the neurogenic niche of the subventricular zone flowing with the CSF to participate in the regulation of the neurogenic niche. As we unfold the multiples trans-fluid talks between discrete brain domains we will have more tools to influence such talks.

## Introduction

The identification of neural stem cells (NSCs) in the adult central nervous system closed down a long-held dogma that neurons are formed exclusively during brain development. The mammalian brain retains the capacity to generate new neurons throughout life in two main locations, the subventricular zone (SVZ) of the lateral ventricles and the hippocampal dentate gyrus (Alvarez-Buylla and Garcia-Verdugo, 2002; Gage, 2002).

The cellular and molecular mechanisms that guide the progression from a dividing NSCs to a functional neuron are far from being understood. A series of components of the neurogenic niche has been identified, including cell–cell interactions, secretory factors, vascular requirements, and specific innervation (Hagg, 2009; Pathania et al., 2010; Faigle and Song, 2013). However, CSF-born signals have largely been overlooked (see below). Key questions remain unsolved. What does control where and how adult neurogenesis occur? Which are the mechanisms and signals underlying neuronal migration, in-fate integration and function? Which are the sources of these signals? How do these signals reach their target?

The design of the CSF-neurogenic niche interphase, i.e., NSC projecting a process to the CSF and bearing a 9+0 cilium, neighboring bi-ciliated and multiciliated cells organized as spatial units around the NSC process (Merkle et al., 2007; Mirzadeh et al., 2008), and the numerous neurotropic, mitogenic, and morphogenic factors, secreted into the CSF, suggest that the CSF should be regarded as a key pathway conveying signals to the pre- and postnatal neurogenic niche. However, this promising research field has largely been neglected. This review aims (1) to bring together early and recent information on the CSF as an integrative pathway; (2) to provide information to understand how the SCO, an ancient brain gland, and other periventricular secretory structures, may contribute to the regulation of embryonic and adult neurogenesis.

## The Cerebrospinal Fluid (CSF), a Pathway for the Delivery of Factors throughout the Brain

The CSF results from the secretion by the choroid plexuses and the bulk flow of the interstitial fluid of brain parenchyma to the ventricles and to the subarachnoid space. In humans, approximately 600 ml of CSF is produced each day. The rate of CSF production displays circadian variations, with lowest levels around 06:00 PM and a nightly peak at about 02:00 AM (Nilsson et al., 1992). The CSF moves along the ventricles and subarachnoid space driven by two mechanisms. The bulk of CSF moves from the main site of origin, the choroid plexus of the lateral ventricles, to the sites of reabsorption. Pulsation of large brain arteries contribute to this bulk flow (Iliff et al., 2013). The laminar flow is a supra-ependymal compartment, about 200 μm thick, where the CSF flow is driven by the cilia beating of multiciliated ependyma (Worthington and Cathcart, 1966; Cifuentes et al., 1994; Siyahhan et al., 2014). Molecular, cell biology and neuroimaging research indicates that CSF physiology is more complex than formerly thought. Aspects now being examined include the various sites of CSF formation and re-absorption, CSF proteomic and the changing CSF composition along its pathway (Brinker et al., 2014; Orešković and Klarica, 2014).

Cerebrospinal fluid proteomics is showing a wealth of over 200 proteins (Zappaterra et al., 2007). A long series of peptides and neurotransmitters are also present in the CSF. Some of these compounds move by bulk flow from the interstitial fluid of brain parenchyma, many are secreted by neurons, glia, and ependyma into the CSF, others are transported by specific transport systems from blood to ventricular CSF (choroid plexus) while a few of them originate from cells present in the CSF.

The CSF is a heterogeneous and highly dynamic compartment that changes its molecular composition as it unidirectionally moves through the various ventricular and subarachnoidal compartments. The choroid plexus of the lateral ventricles, the interstitial fluid of the parenchyma surrounding these ventricles and axons endings secreting into these cavities are the source of molecules forming this “first” fluid. At the third ventricle new compounds are added to the CSF by hypothalamic neurons, the pineal gland and the local choroid plexus (Rodríguez, 1976; Nicholson, 1999; Johanson et al., 2008). When entering the Sylvius aqueduct the CSF is enriched by the secretion of the SCO (Vío et al., 2008). Consequently, the CSF of the fourth ventricle is different as compared to that of the lateral ventricles (Zappaterra et al., 2007). This partially explains the different protein composition between the CSF collected from the lateral ventricles and that obtained from a subarachnoid compartment (Vío et al., 2008). Furthermore, at the interphase brain parenchyma/subarachnoid space there is a bidirectional flow of CSF and interstitial fluid along the large paravascular spaces that surround the penetrating arteries and the draining veins. Since water movement along this pathway is mediated by astroglial aquaporin-4 water channels, this paravascular pathway has been termed “glymphatic system” (Iliff et al., 2012, 2013). This pathway facilitates efficient clearance of interstitial solutes and its failure may lead to neurodegeneration (Iliff et al., 2015).

**FIGURE 1:**
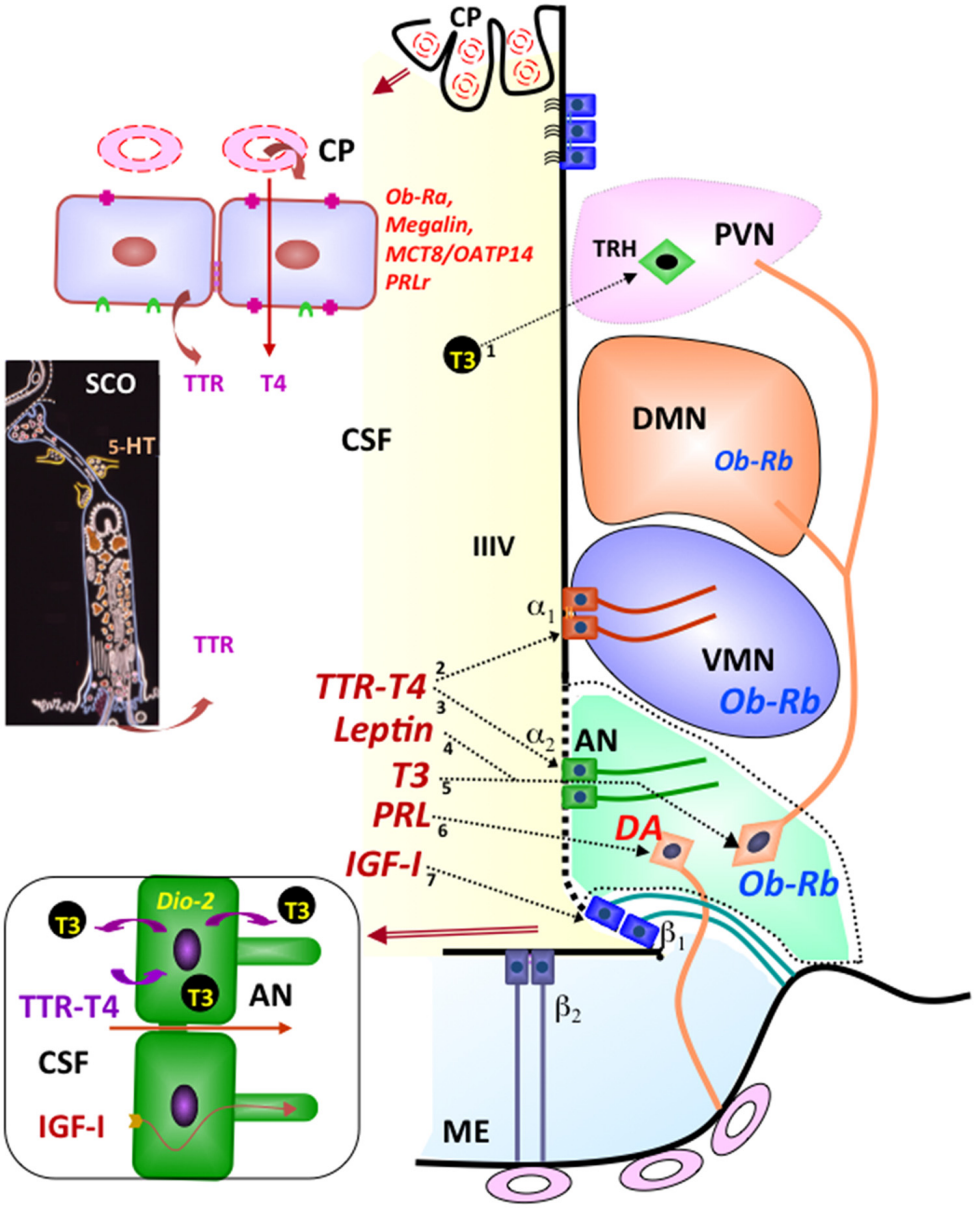
**Integrative pathways involving the CSF.** By receptor mediated transport at the choroid plexus (CP), leptin (Ob-Ra), insulin growth factor I (megalin), thyroid hormones (MCT8/OATP14), and prolactin (PRLr) are transported from blood to CSF. Transthyretin (TTR) is secreted by choroid plexus and the subcommissural organ (SCO) into the CSF. The secretory activity of the SCO is under serotonin (5-HT) inhibitory control. Most CSF T4 is bound to TTR. TTR-T4 complexes are taken up by tanycytes that express deiodinase 2 (arrows 2, 3). Here (bottom left panel), T4 is converted to T3 and then released into the intercellular space of the arcuate nucleus (arrow 5) or into the CSF to reach the TRH-parvocellular neurons of the paraventricular nucleus (arrow 1). The milieu of the arcuate nucleus (AN; green background) is especially exposed to molecules present in the CSF and closed to the median eminence (ME) and ventromedial nucleus (VMN). Leptin present in the CSF may readily reach the neurons expressing the Ob-Rh receptor of the arcuate (arrow 4), ventromedial and dorsomedial nuclei of the hypothalamus. CSF prolactin (arrow 6) may reach the dopamine-secreting neurons (DA) of the arcuate nucleus that project to the portal capillaries of the median eminence (light-blue background). CSF insulin growth factor I (arrow 7) is internalized by β tanycytes and transported along their processes. Modified after Rodríguez et al. (2010).

The long series of biologically active proteins, peptides, and neurotransmitters present in the CSF reach this fluid through different mechanisms. (1) Neurotransmitters and their metabolites reach the CSF via the bulk flow of parenchymal fluid. (2) Regulated secretion into the CSF of biologically active compounds by the circumventricular organs (SCO, pineal gland, choroid plexuses, and median eminence), such as SCO-spondin, basic FGF, melatonin, TTR, TTR-T4 complex, TTR-T3 complex, nerve growth factor (NGF), transforming growth factor-β (TGFβ), vascular endothelial growth factor (VEGF), transferrin, and vasopressin (Gross, 1987; Johanson et al., 2008; Rodríguez et al., 2010; Johansson, 2014; **Figure 1**). (3) Selective and circadianly regulated secretion by CSF-contacting neurons of serotonin and neuropeptides such as vasopressin, oxytocin, and somatostatin (Rodríguez, 1976; Vigh-Teichmann and Vigh, 1989; Vígh et al., 2004). (4) Transport of peripheral hormones through the choroid plexus. Most of the transported hormones, such as leptin, prolactin, and thyroxin have specific targets, mostly the hypothalamus (Chodobski and Szmydynger-Chodobska, 2001; Rodríguez et al., 2010; **Figure 1**). Furthermore, recent findings indicate that cells forming the ventricular walls release into the CSF microvesicles containing signaling and intracellular proteins (Marzesco et al., 2005; Street et al., 2012; Chiasserini et al., 2014; Feliciano et al., 2014).

Thus, the early view that the CSF is a medium carrying brain-borne and blood-borne signals to distant targets within the brain (Rodríguez, 1976) has largely been supported by numerous investigations (Wood, 1983; Johnson and Gross, 1993; Johanson et al., 2008; Rodríguez et al., 2010). Worth mentioning here is the much neglected system of CSF-contacting neurons most likely playing receptive functions sensing CSF composition. Most of these neurons are bipolar with the dendritic process reaching the CSF and endowed with a 9+0 single cilium (Vígh et al., 2004; **Figure 4D**).

## The Subcommissural Organ

**FIGURE 2:**
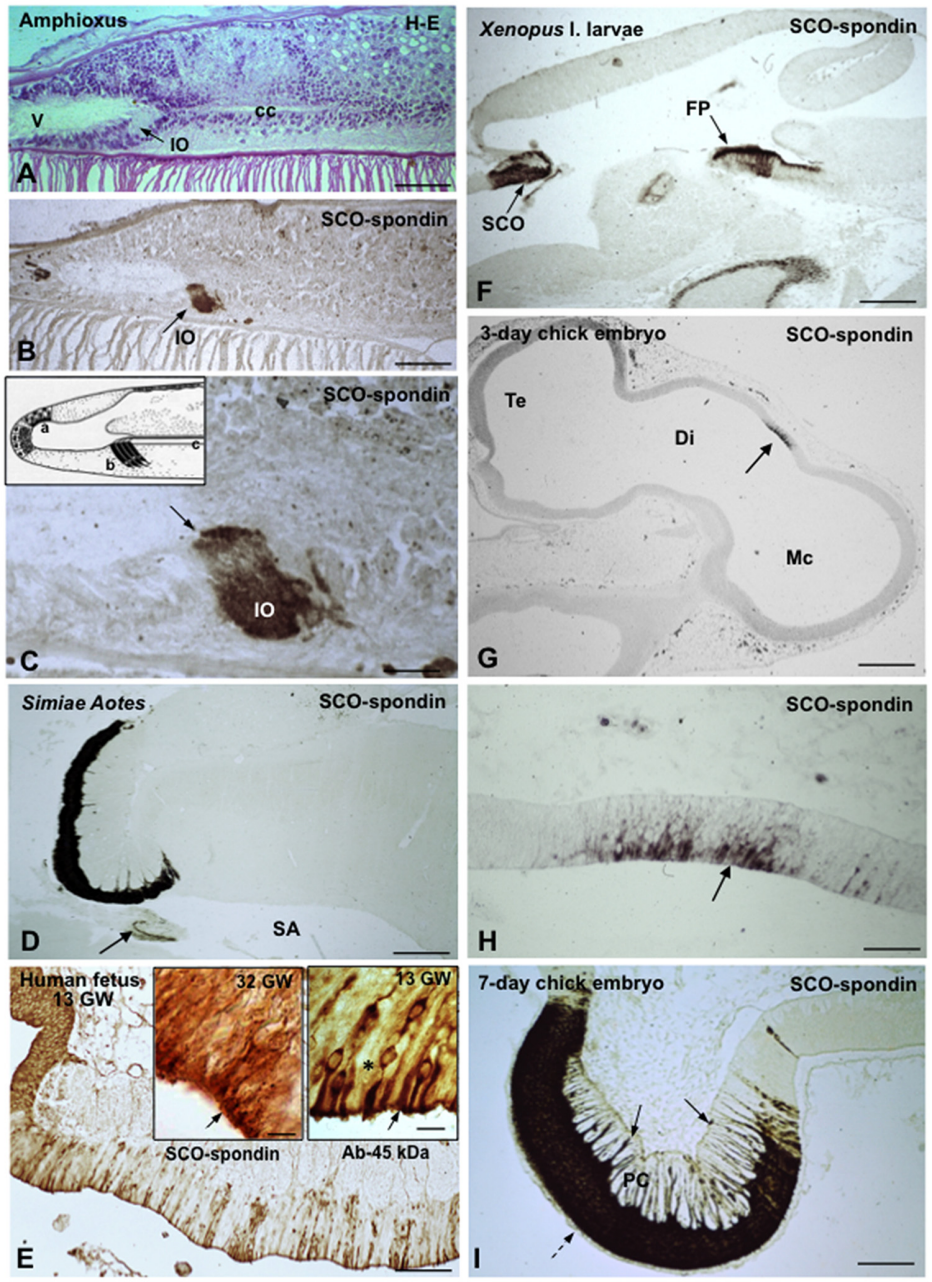
**The subcommissural organ is the phylogenetically oldest brain gland and the first to differentiate in ontogeny. (A–E)** From amphioxus to primates, 500 million years of evolution. **(A–C)** Sagittal sections through the CNS of the amphioxus (*Branchiostoma lanceolatum*, Acrania), showing the location **(A)** and immunoreactivity **(B,C)** of the cells forming the Infundibular organ (IO). V, ventricle; cc, central canal; from Olsson et al. (1994). **(C)** Line drawing of the CNS of the amphioxus showing a secretory ependyma in the recessus neuroporicus (a), the infundibular organ (b) and the central canal with Reissner fiber (c); from Olsson and Wingstrand (1954). **(D)** Subcommissural organ and Reissner fiber (arrow) of the primate Aotes. SA, sylvius aqueduct; from Rodríguez et al. (1993). **(E)** Sagittal section through the epithalamus of a 13-weeks-old human fetus immunostained with an antiserum against a 45 kDa compound (most likely corresponding to TTR) obtained from the CSF of a hydrocephalic fetus. A population of ependymocytes are strongly immunoreactive; from Rodríguez et al. (1993). **Right inset** detailed magnification of previous figure showing immunoreactive (arrow) and immunonegative (asterisk) ependymal cells; **left inset** SCO from a 32 GW fetus immunostained for SCO-spondin; all cells are immunoreactive (arrow). **(F)** Sagittal section through the CNS of a *Xenopus* l larvae. The cells of the subcommissural organ (SCO) and the floor plate (FP) strongly express SCO-spondin. **(G)** Sagittal section through the CNS of a 3-days-old chick embryo. A small group of neuroependymal cells located a the roof of the diencephalic vesicle (Di) expresses SCO-spondin (arrow). Te, telencephalon; Mc, mesencephalon. **(H)** Detailed view of previous figure showing that SCO-spondin is mainly located in the apical region of the neuroependymal cells (arrow). **(i)** At the 7th day of incubation, the chick SCO is fully differentiated with SCO-spondin located in the cell body of ependymocytes (broken arrow) and along their basal processes ending at the pial membrane (full arrows). PC, posterior commissure; from Schoebitz et al. (1986). Scale bars: **(A,B)** 80 μm; **(C)** 16 μm; **(D)** 400 μm; **(E)** 100 μm; right Inset 9 μm; left inset 8 μm; **(F)** 300 μm; **(G)** 280 μm; **(H)** 56 μm; **(I)** 85 μm.

The SCO is an ancient and highly conserved brain gland present throughout evolution of chordates, from amphioxus (Rodríguez and Oksche, 1993; Olsson et al., 1994) to man (Rodríguez et al., 2001; **Figures 2A–E**). The astonishing amphioxus, an evolutionary leap made at the bottom of the ocean over 500 million years ago, already has a small group of cells secreting a very thin Reissner fiber (RF) (Olsson and Wingstrand, 1954; **Figure 2C**, inset) that immunoreacts with antibodies against mammalian SCO-spondin (Olsson et al., 1994). The ancient SCO-spondin-secreting cells symbolize a family resemblance between amphioxus and primates (compare **Figures 2B,D**). SCO-spondin could be considered a member of an exclusive group of proteins accompanying the brain through its long lasting evolution what, in turn, highlight the functional significance of this molecule.

In ontogeny, the SCO is one of the first brain structure to differentiate (Schoebitz et al., 1986; **Figures 2F–I**). In the human, the SCO can be morphologically distinguished in 7-weeks-old embryos. By the 13th gestational week (**Figure 2E**), the SCO is a fully differentiated gland that remains secretory active throughout the fetal life, releasing CSF-soluble proteins (Rodríguez et al., 2001). During childhood the secretory parenchyma of the SCO is confined to islets of secretory ependymal cells. In non-human species, the SCO is a highly differentiated gland during most of the fetal period and throughout life span (Rodríguez et al., 1984a; Schoebitz et al., 1986, 1993; **Figure 2D**).

**FIGURE 3:**
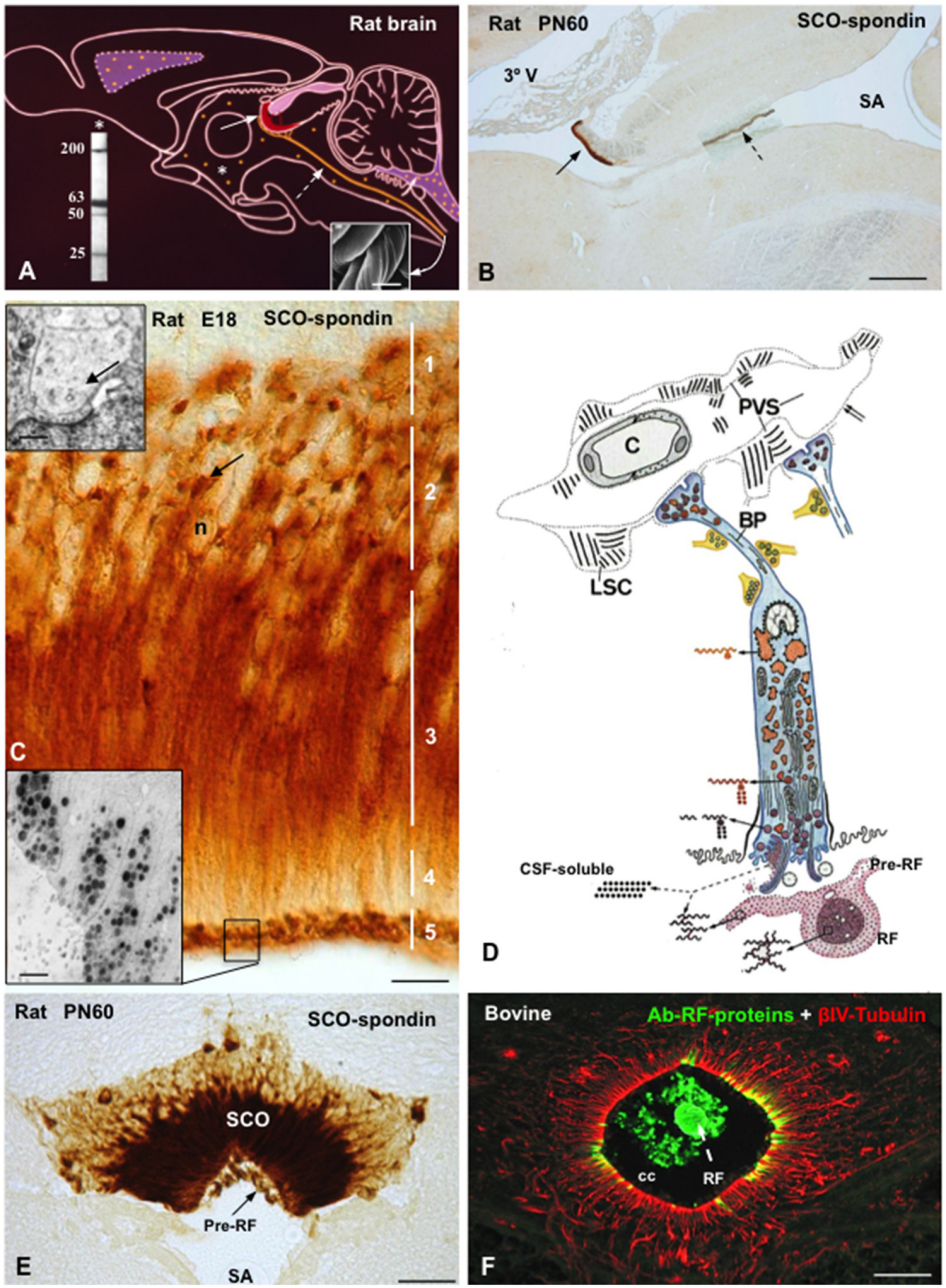
**The subcommissural organ-Reissner fiber complex. (A)** Drawing depicting the rat subcommissural organ (red, full arrow)- Reissner fiber (orange, broken arrow) complex and the CSF-soluble secretion (orange dots, asterisk). (Right inset) Scanning electron microscopy of bovine RF collected from the central canal. Left inset. Western blot of CSF of PN30 rats, immunoreacted with antibodies against SCO-spondin. CSF-soluble compounds of 200, 63, 50, and 25 kDa of molecular weight are shown. **(B)** Sagittal section of a rat brain immunostained with anti-SCO-spondin at postnatal day 60. The SCO (full arrow)-RF (broken arrow) complex is selectively immunoreactive. **(C)** High magnification of the SCO of a rat embryo (E18) immunostained with anti-SCO-spondin. Zonation of a SCO-cell (1–5) is shown. Arrow points to paranuclear immunoreactive masses corresponding to RER. Upper inset. Electron microscopy of dilated RER cisternae (arrow). Lower inset. Electron microscopy immunocytochemistry using anti-SCO spondin showing secretory granules stored at the apical cell pole. **(D)** Drawing depicting the ultrastructure and the secretory process of a SCO-ependymal cell. They are bipolar cells, with and apical pole contacting the ventricular CSF and a basal process projecting to local capillaries and to the subarachnoid space. Glycoproteins secreted by the SCO cells either remain soluble into CSF or polymerize forming the RF. The secretory material upon release condenses, first as a film on the surface of the organ (pre-RF) and, after further packaging, into RF. The basal processes of ependymal cells (BP) receive abundant serotonergic, gabaergic, and catecholaminergic neural inputs and end on a network of basal lamina containing long spacing collagen (LSC). PVS, perivascular space. **(E)** Frontal section of a rat brain at PN60 immunostained with anti-SCO-spondin. SCO and pre-RF are strongly reactive. **(F)** Frontal section of the bovine spinal cord processed for double immunofluorescence using anti-RF proteins (green) and βIV-tubulin (red). The central canal (cc) contains Reissner fiber (RF, green) and is lined by tanycytes-like ependymal cells (red). Scale bars: **(B)** 200 μm; **(C)** 10 μm; **(E)** 40 nm; **(F)** 20 μm. From Rodríguez et al. (1993); Vío et al. (2008), Ortloff et al. (2013).

The SCO is located in the dorsocaudal region of the third ventricle, at the entrance of the Sylvian aqueduct (**Figures 3A,B**). The secretory cells of the SCO are arranged into two different layers, the ependyma and the hypendyma.

The ependymal cells of the SCO are bipolar, with and apical pole contacting the ventricular CSF and a basal process projecting to local capillaries and to the subarachnoid space (Leonhardt, 1980; Rodríguez et al., 1992, 2001; **Figure 3D**). The cell body presents a clear zonation, which has facilitated the investigation of the secretory process. Different phases of this process occur in discrete but separate areas of the cell, namely, (1) synthesis in the perinuclear and intermediate regions, (2) storage of precursor forms in big RER cisternae located in the subnuclear region, (3) processing and packaging in the intermediate region, (4) transport in the subapical region, (5) storage of processed forms and release in the apical cell pole (Rodríguez et al., 1992, 2001; **Figures 3C,D**). Further, the SCO offers a unique feature: the secretory material upon release condenses, first as a film on the surface of the organ and then, after further packaging, into RF (**Figures 3D–F**). Most of the ultrastructural characteristics of the hypendymal cells are similar to those described for the ependymal cells.

In non-mammalian species all ependymal cells of the SCO display long and slender processes that traverse the posterior commissure and end on the external basement membrane of the brain (**Figure 2I**). Their terminals are loaded with secretory granules. The most likely fate of this secretion is the local leptomeningeal cistern (there is no continuous subarachnoid space in non-mammalian species). In mammals, the basal processes of the SCO cells containing secretory granules either project to the subarachnoid space or to the subependymal capillaries. Here, the processes end on a network of extensions of the perivascular basement membrane formed by long-spacing collagen, a unique arrangement and a landmark of the SCO (Rodríguez et al., 1992, 2001). The basal processes of ependymal and hypendymal cells receive abundant synaptic contacts of various nature (see innervation below; **Figure 3D**).

The whole arrangement of the SCO cells indicates that (i) they secrete compounds to the ventricular CSF, the subarachnoidal CSF and probably to blood; (ii) this secretory activity is under neural control. The nature of the compounds secreted into ventricular CSF is only partially known (i.e., SCO-spondin, TTR and probably basic FGF), whilst that of the compounds contained in the secretory granules stored at the perivascular and subarachnoidal ependymal terminals is unknown.

In most circumventricular organs the blood-brain-barrier has been displaced from the vascular side to the ependymal side so that they are open to blood and tightly closed to both the CSF and the neighboring neural parenchyma (see Rodríguez et al., 2010). Due to the design of its barriers, the SCO is closed to blood and to the CSF, becoming a sort of an *island within the brain* (Rodríguez et al., 1992, 1998). The functional meaning of this unique arrangement is unknown.

## The Secretory Products of the Subcommissural Organ

The SCO secretes into the ventricular CSF two classes of proteins, the ones that remain soluble in the CSF and that, consequently, go with the flow and those that aggregate to form an insoluble, ever-growing structure, the RF (**Figures 3A,B**).

### RF-Glycoproteins

The ependymal cells secrete *N*-linked glycoproteins of high molecular mass that, upon release undergo a progressive packaging until forming a fully packaged RF in the postnatal life (Sterba, 1969; Nualart et al., 1991). By addition of newly released glycoproteins to its proximal end, RF grows caudally and extends along the aqueduct, fourth ventricle, and the whole length of the central canal of the spinal cord (Sterba, 1969; Leonhardt, 1980; Caprile et al., 2003; **Figures 3B,D,F**). RF material continuously arrives at the dilated caudal end of the central canal, known as the terminal ventricle or ampulla, where RF-glycoproteins undergo chemical modifications (loss of sialic acid residues), disaggregate and then escape through openings in the dorsal wall of the ampulla to finally reach local vessels (Olsson, 1958; Peruzzo et al., 1987; Rodríguez et al., 1987).

### SCO-Spondin

Molecular procedures have led to the identification of SCO-spondin as a multidomain, large-molecular mass glycoprotein (540 kDa) secreted by the SCO into the ventricular CSF, where it contributes to form the RF (Nualart et al., 1991; Gobron et al., 1996; Meiniel, 2001; see further below; **Figures 3A,B**). At variance with SCO-spondin forming RF, there are compounds of 200, 63, 50, and 25 kDa molecular mass that are consistently found in the CSF of rodents (Vío et al., 2008) and humans (**Figure 3A**, inset). These compounds react with specific antibodies against SCO-spondin and most likely result from a further processing of SCO-spondin. We regard these proteins as CSF-soluble SCO-spondin-derived compounds. In adulthood, the CSF contains both RF-SCO-spondin and the soluble SCO-spondin related compounds (Vío et al., 2008). During the embryonic period, the very active SCO of all species studied (**Figure 2I**), including the human (**Figure 2E**, left inset), secretes CSF-soluble SCO-related proteins while RF is missing (Rodríguez et al., 1998, 2001; Hoyo-Becerra et al., 2006; Vío et al., 2008).

At early developmental stages SCO-spondin is also expressed by the floor plate cells that release it into the fetal CSF and also transport it along their basal processes (paracrine effect?; Yulis et al., 1998; Richter et al., 2001; **Figure 2F**). The floor plate, a key structure in brain development, participates in the neural patterning and axon guidance of the ventral neural tube.

### Transthyretin

Transthyretin, a protein involved in the transport of thyroid hormone and retinol in the CSF (Chanoine and Braverman, 1992; Bernal, 2002), is expressed by the ependymal cells of the SCO (Montecinos et al., 2005). The mRNA encoding TTR and the 14 kDa protein are expressed in the SCO under *in vivo* and *in vitro* conditions. Organ cultured SCO secretes TTR into the culture medium, indicating that the SCO synthesizes TTR and secretes it into the CSF (Montecinos et al., 2005). The SCO possesses two populations of secretory cells, one secreting both RF-glycoproteins and TTR and the other secreting only the former (**Figures 2E and 8H**). TTR was detected in the SCO of bovine embryos and human embryos (**Figure 2E**) suggesting that this ependymal gland is a source of TTR during brain development SCO (Montecinos et al., 2005).

### Other Proteins

Antibodies raised against “CSF-specific” glycoproteins (glycoproteins present in the CSF but missing from the plasma) obtained from the CSF of hydrocephalic children react with the human and rat SCO (Rodríguez et al., 1993, 2001; Montecinos, 1995). Immunoreactive-basic fibroblast growth factor (bFGF) has been also detected in the SCO (Cuevas et al., 1996).

The detection in the CSF of the lateral ventricle and cisterna magna of CSF-soluble compounds secreted by the SCO (Rodríguez et al., 1993; Vío et al., 2008) indicates that such a material circulates in the ventricular and subarachnoidal CSF (**Figure 3A**). Because both CSF compartments are in open communication with the brain tissue, the SCO-soluble secretion could reach any region of the central nervous system, with the exception of the other circumventricular organs that have a tight barrier with the CSF.

The secretory activity of the SCO is under neural control. This include serotonergic (Bouchaud, 1979; Jiménez et al., 2001), gabaergic and catecholaminergic (Balaban et al., 1994; Tomé et al., 2004) inputs (**Figure 3D**). SCO-cells also express receptors for angiotensin II (Ghiani et al., 1988), endothelin 1 and bradykinin (Schöniger et al., 2009). The serotonergic input exerts and inhibitory control on the expression and release of SCO-spondin (Richter et al., 2004).

## The Cerebrospinal Fluid, the Subcommissural Organ, and the Neurogenic Niche

**FIGURE 4:**
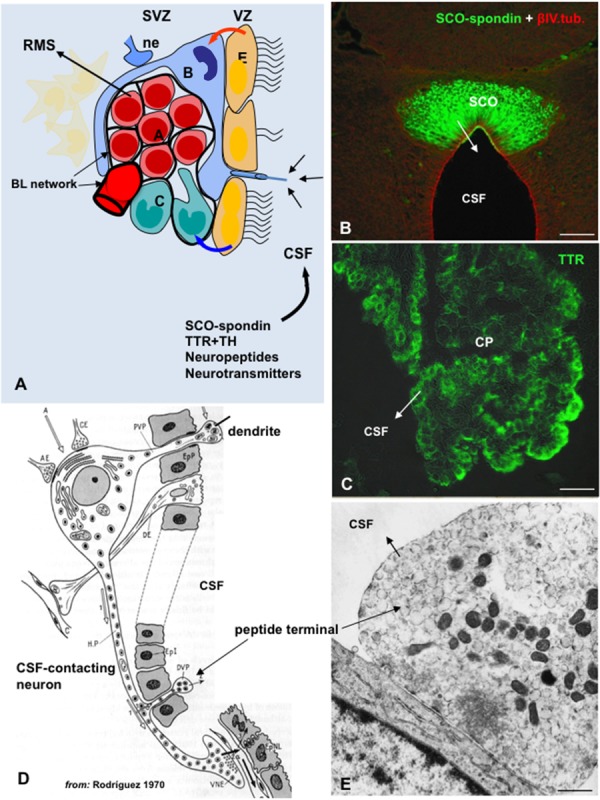
**The cerebrospinal fluid is a pathway for the delivery of neurotropic factors to the adult SVZ niche. (A)** Cell organization of SVZ niche in the adult brain. SVZ astrocytes (**B**, blue) are stem cells which generate migrating neuroblasts (**A**, red) destined for the olfactory bulb via rapidly dividing transit-amplifying cells (**C**, green). A specialized basal lamina (BL, black) extends from perivascular cells and contacts all cell types, including multiciliated ependyma cells (**E**, orange). Ependymal cells, neural terminals (ne), the extracellular matrix (ECM)-basal lamina (BL) network, and the cerebrospinal fluid (CSF) are key components of the niche and regulator of the adult neurogenesis. Stem cells display a single 9+0 cilium to sensor CSF signals. Compounds secreted into the CSF by circumventricular organs such as the subcommissural organ (SCO) and choroid plexus (CP), or by CSF-contacting neurons can readily reach the SVZ (modified after Riquelme et al., 2008). **(B)** Frontal section of the rat SCO immunostained with antibodies against SCO-spondin and βIV-tubulin (from Ortloff et al., 2013). **(C)** Choroid plexus immunostained for TTR. **(D)** Drawing depicting a hypothalamic peptidergic CSF-contacting neuron with a dendrite projecting to the ventricle bearing a 9+0 cilium, and axon projecting to the capillaries of the pituitary gland and bearing and axonal branch reaching the ventricle (from Rodríguez, 1976). **(E)** Electron microscopy of a peptide terminal within the ventricle, with neurosecretory granules undergoing exocytosis. Scale bars: **(B)** 120 μm; **(C)** 35 μm; **(E)** 700 nm.

All cells forming the central nervous system are generated from a common source, neuroepithelial/NSCs located in the ventricular zone (VZ) of the developing brain. After birth, and during life span, neurogenesis continues at specific brain areas, known as neurogenic niches. Adult neurogenesis is mostly confined to two brain regions, the SVZ of the lateral ventricles (**Figure 4A**) and the subgranular zone (SGZ) of the hippocampal dentate gyrus (Alvarez-Buylla and Garcia-Verdugo, 2002; Gage, 2002). Several publications have also reported the generation of new neurons in other regions of the adult brain, including the neocortex, the amygdala, the hypothalamus, the circumventricular organs, the striatum and the substantia nigra (Dellmann and Rodríguez, 1970; Bennett et al., 2009; Migaud et al., 2010; Furube et al., 2015).

The molecular mechanisms that control neurogenesis are being extensively studied (reviewed by Urban and Guillemot, 2014). It is becoming evident that NSCs of the embryonic and adult brain are not as multipotential as previously thought. Instead, subpopulations of NSCs appear to be committed to generate specific types of neural cells (Alvarez-Buylla et al., 2008; Taverna et al., 2014). The mechanisms underlying the NSCs heterogeneity are among the most exciting questions in the field (DeCarolis et al., 2013; Encinas et al., 2013; Giachino et al., 2014). Neurogenesis involves several steps such as proliferation, commitment of the new cells to a neuronal phenotype, their migration and maturation and, finally, the establishment of appropriate synaptic contacts (Abrous et al., 2005; Braun and Jessberger, 2014). These steps are regulated by intrinsic and extrinsic factors. Intrinsic factors include cell-to-cell interactions and niche-derived morphogens released by stem cells, ependyma cells, and endothelial cells (**Figure 4A**); extrinsic factors include signals generated in the vicinity of the niche as well as blood-borne and CSF-borne compounds (Sawamoto et al., 2006; Riquelme et al., 2008; Hagg, 2009; Pathania et al., 2010; Faigle and Song, 2013; **Figures 4A–E**).

The NSCs of the embryonic VZ are characterized by projecting a 9+0 single cilium to the fetal CSF (Sotelo and Trujillo-Cenóz, 1958; Tramontin et al., 2003). There is evidence that molecules present in the fetal CSF are cues for the NSCs (Parada et al., 2006; Zappaterra et al., 2007) and that receptors for insulin and insulin-like growth factors 1 and 2, FGF, sonic hedgehog and BMP, localize at the apical plasma membrane (Lehtinen and Walsh, 2011). Similar to the embryonic NSCs, the NSCs of the adult SVZ project a process that reaches the ventricular CSF and bears a single 9+0 cilium (Doetsch et al., 1999). Although virtually nothing is known about the molecular characteristic of this cilium, it seems most likely that it is receptive to signals present in the fetal and adult CSF (**Figure 4A**). Interestingly, primary cilia ablation leads to disruption of hedgehog signaling which plays key roles in brain development and in adult neurogenesis (Tong et al., 2014).

Cerebrospinal fluid-long-distance cues may act directly on NSC and progenitor cells to regulate neurogenesis (Johanson et al., 2008; Johansson, 2014). Many of the CSF compounds secreted by the CSF-contacting neurons and circumventricular organs, such as the SCO and the choroid plexuses, are good candidates to signal the receptive “CSF-contacting NSCs” of the SVZ niche (**Figures 4B–E**). The design of the CSF-neurogenic niche interphase and the numerous neurotropic factors secreted into the CSF, point to the CSF as a key milieu for the SVZ niche. A further thought concerns the properties of the CSF–SVZ barrier. Neither the cell junction complexes between the different component of the ependymal component of the niche (NSC processes, bi- and multi-ciliated ependymal cells) nor the barrier properties of this cell layer have been properly investigated. This information is required for a better understanding of the relationships between the processes taking place in the SVZ and, via the CSF, in other brain regions.

## Effects of SCO-Compounds on Fetal Neurogenesis

The fetal CSF may be regarded as the main component of the milieu of stem cells and progenitor cells of the germinal zone providing signals participating in embryonic brain growth and differentiation (Miyan et al., 2003; Gato and Desmond, 2009; Gato et al., 2014). Quality and quantity of proteins of fetal CSF vary throughout development (Mashayekhi et al., 2002; Zappaterra et al., 2007; Vío et al., 2008), and differ from those of adult CSF (Vío et al., 2008). In all species, including the human, the SCO secretes CSF-soluble proteins during most of the fetal period. SCO-spondin, SCO-spondin-derived polypeptides, TTR and other detected but not-yet identified secretory compounds are released by the ependymal cells of the SCO into the ventricular CSF, while the secretory hypendymal cells secrete into the subarachnoid space a material reacting with antibodies against RF-glycoproteins and likely corresponding to SCO-spondin-derived compounds (Rodríguez et al., 1984a,b, 1993; Schoebitz et al., 1993; Hoyo-Becerra et al., 2006; Vío et al., 2008). Eight bands immunoreacting with antibodies against RF-glycoproteins are consistently found in CSF samples from rats at E18, E20, and PN1. Only four of these compounds are detected in the CSF of PN30 rats, indicating that secretion and/or processing of SCO secretory proteins in the fetal period is different from that of adult life (Vío et al., 2008).

Subcommissural organ-spondin, promotes neuronal growth and differentiation during the embryonic development (Monnerie et al., 1995; Gobron et al., 2000; Meiniel, 2001; Stanic et al., 2010; Grondona et al., 2012; Vera et al., 2013). In chick embryos, SCO-spondin is released into the embryonic CSF at early stages of development (Schoebitz et al., 1993; Hoyo-Becerra et al., 2006). Inhibition of SCO-spondin by injecting antibodies into the embryonic CSF or using shRNA to knockdown this protein drastically decreases the neurodifferentiation process (Vera et al., 2013). This effect appears to be mediated by interaction of SCO-spondin with low density lipoproteins from embryonic CSF (Vera et al., 2015). During the fetal period, the basal route of secretion of the SCO via the processes of the hypendymal cells is more developed than in the postnatal period (Schoebitz et al., 1986; **Figure 2I**). There is evidence that SCO-spondin is released from these processes becoming part of the ECM (Caprile et al., 2009) contributing to the organization of the axons forming the posterior commissure (Stanic et al., 2010; Grondona et al., 2012). This effect appears to be mediated by the interaction of SCO-spondin with β1-integrin (Caprile et al., 2009).

After early studies had shown that insufficient thyroid hormone supply to the brain leads to neurodevelopmental defects and mental retardation (revised by Morreale de Escobar, 2001), the effects of thyroid hormones on brain development have been thoroughly investigated. Transthyretin (TTR), secreted by the choroid plexus (Dickson et al., 1986; Buxbaum and Reixach, 2009; Johansson, 2014) and the SCO (Montecinos et al., 2005) in ontogeny, is a CSF protein delivering thyroid hormones and retinol to areas involved in pre- and postnatal neurogenesis (Chanoine and Braverman, 1992; Kassem et al., 2006; Richardson et al., 2007; Alshehri et al., 2015). It is worth noting that TTR is not essential for thyroid hormones distribution to most tissues in adult mice, one notable exception being the SVZ of the brain (Monk et al., 2013). Here, thyroid hormones regulate the cell cycle of NSC and neural progenitor cells by influencing both proliferation and apoptosis (Lemkine et al., 2005; Richardson et al., 2007). Further, T3 exerts a role in NSC commitment toward neuroblasts (Kapoor et al., 2012; López-Juárez et al., 2012). T4 and T3 might also influence oligodendroglial differentiation (Almazan et al., 1985; Franco et al., 2008; Fernández et al., 2009).

The proteomic screening of CSF has revealed differences in the CSF proteins of non-affected and hydrocephalic rats, in particular with respect to SCO-secretory proteins and TTR (Ortloff et al., 2013). TTR concentration is higher; it is speculated that it would be involved in neuroprotection. In addition, immature forms of SCO-spondin and SCO-spondin related compounds have been detected into the hydrocephalic CSF (Ortloff et al., 2013). Such an abnormal CSF plays a role in the deficient cortical development of this mutant (Mashayekhi et al., 2002). Recent findings in HTx rats and hydrocephalic human fetuses strongly indicate that hydrocephalus and abnormal neurogenesis are two inseparable phenomena (Guerra et al., 2015).

## Effects of SCO-Compounds on Adult Neurogenesis

In all species but human (see above), the SCO remains highly differentiated and secretory active through life span (Rodríguez et al., 1984a). During this long period, the SCO continues to secrete SCO-spondin, SCO-spondin-derived compounds and TTR. The latter two are CSF-soluble and go with the CSF flow. What is the fate and target of these compounds in the adult brain? Would their early neurogenetic properties also be expressed in adulthood? The evidence collected during recent years points to a positive answer (see below).

### Basic Fibroblast Growth Factor

Multiple studies demonstrate the important role of bFGF in regulating neurogenesis and mediating brain repair processes. bFGF has been shown to be a potent mitogenic factor for NSC and progenitor cells both *in vitro* and *in vivo* (Gage et al., 1998; Wagner et al., 1999; Cheng et al., 2001). Evidence indicates that bFGF exerts proliferative effects on quiescent NSC (Zheng et al., 2004; Wang et al., 2011).

### Transthyretin

Transthyretin synthetized by the choroid plexus and the SCO is secreted into the CSF. A marked difference between these two sources of TTR is that the SCO cells, at variance with the choroidal cells, are not open to the blood stream and their secretory activity is under the control of a complex neural input (**Figure 1**). Within the choroidal cells, TTR binds thyroxin (T4) that has entered these cells either by passive diffusion or by specific transporters (Alshehri et al., 2015). Via the CSF, the TTR-T4 complexes are carried to specific brain areas (**Figure 1**).

T4 is the predominant iodothyronine in plasma. However, T3 is the major receptor-active form of thyroid hormones. Consequently, T4 has to be converted by the effect of diodinase 2 into T3. The conversion of thyroxin present in the CSF into T3 takes place, exclusively, in the tanycytes located in the hypothalamus (Lechan and Fekete, 2005, 2007; Rodríguez et al., 2005, 2010). Tanycytes are virtually the only cell type exposed to the CSF that expresses diodinase 2 (Guadano-Ferraz et al., 1997; Diano et al., 2003; Lechan and Fekete, 2005, 2007). Tanycytes take up T4-TTR and/or T4 from the CSF and pour T3 back to the CSF where it forms T3-TTR. The T3-TTR complex has receptors at specific brain regions (Rodríguez et al., 2010; **Figure 1**).

These findings point to a functional relationship, via the CSF, between three different types of ependymal cells, namely, the ependymocytes of the SCO, the choroidal cells of the choroid plexus and tanycytes. The outcome of such an association is to provide signals to the neurogenic niche (**Figure 1**).

### SCO-Spondin and SCO-Spondin-Derived Compounds

**FIGURE 5:**
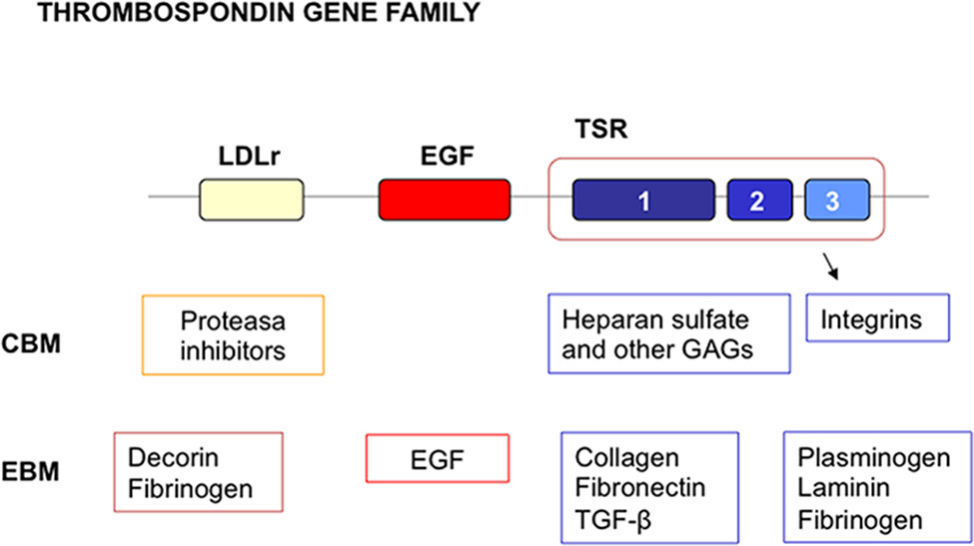
**Multidomain organization of thrombosponin type 1 molecules.** LDL receptor domains are indicated by the yellow box. EGF like domains are indicated by the red box. Thrombospondin types 1, 2, and 3 repeats (TSRs) are indicated by the blue boxes. A number of cellular and extracellular binding molecules for the domains have been identified. Many of these are components of ECM. CBM, cellular binding molecules; EBM, extracellular binding molecules.

**FIGURE 6:**
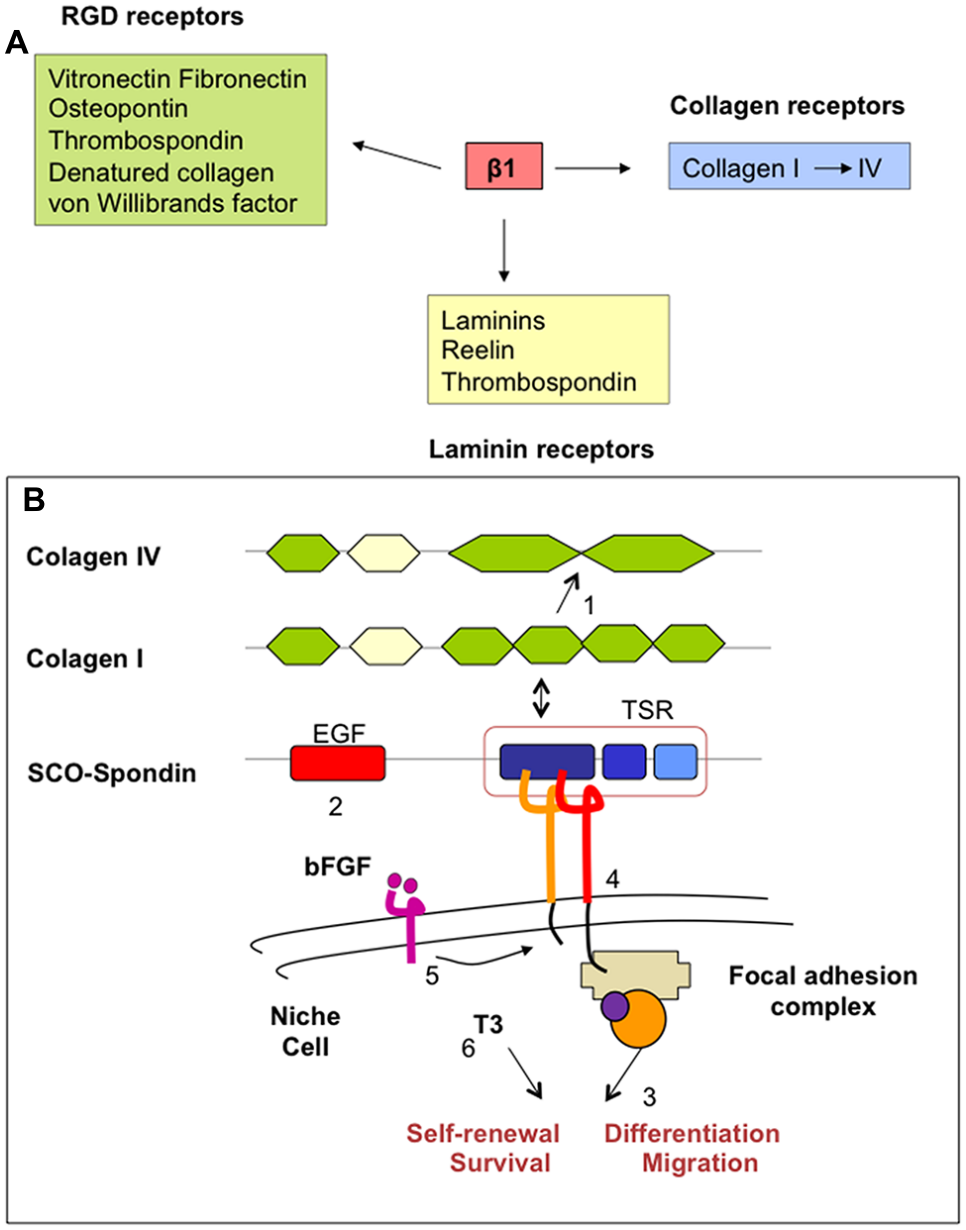
**(A)** Ligands for integrin-β1 heterodimers. Many of these ligands are components of ECM. **(B)** Simplified schematic drawing of how SCO-spondin might promote neurogenesis in the adult SZV niche. SCO-spondin (1) may change the composition of ECM (i.e., transforming the type of collagen) and (2) the availability of growth factors in the niche, modifying (3) the immediate microenvironment and behavior of niche cells. Some of these functions could be mediated (4) by interaction of SCO-spondin with integrin-β1 signaling and (5) cross talking with other essential pathways, like those regulated by bFGF and TTR/thyroid hormones (6).

The complex multidomain organization of SCO-spondin allow to speculate about probable mechanism(s) by which SCO-spondin and SCO-spondin-derived compounds would promote neurogenesis in the adult SVZ niche. This protein displays a unique arrangement of several conserved domains, including 26 thrombospondin type 1 repeats (TSRs), 9 low density lipoprotein receptor (LDLr) type A domains, 2 epidermal growth factor (EGF) like domains, and NH2 and COOH von Willebrand cysteine-rich domains (vWD; Meiniel et al., 2008). All these consensus sequences represent potential sites of protein–protein interaction. Potential binding sites to proteoglycans and growth factors have also been identified (Gobron et al., 2000; Meiniel, 2001; **Figure 5**). Due to the large number of TSR, SCO-spondin is regarded as an extra cellular matrix-like protein belonging to the TSR superfamily. It is involved in multiple functions including cell attachment, motility, proliferation, cell–cell contact, cell aggregation and angiogenesis, all of which are thought to contribute to vascular homeostasis and brain functions (Adams, 2001; Tucker, 2004). This is consistent with the role of SCO-spondin to promote cell differentiation and neurite outgrowth of various neuronal cell populations in cell culture (Monnerie et al., 1995; Meiniel et al., 2003), and the proposed role of SCO-spondin in the formation of posterior commissure during the embryonic development (Stanic et al., 2010; Grondona et al., 2012). Interestingly, through TSR motifs SCO-spondin could bind β1-integrin (**Figure 6A**). This interaction may be essential for the neurite outgrowth induced by SCO-spondin *in vitro* (Bamdad et al., 2004) and for the posterior commissure development *in vivo* (Caprile et al., 2009; Grondona et al., 2012).

In the adult SVZ niche, β1-integrin is highly expressed by NSC, progenitor cells, neuroblasts, and endothelial cells (Shen et al., 2008). Here, integrins provide NSC the capacity to regulate their responsiveness to growth factors (Fuchs et al., 2004; Campos, 2005). Furthermore, β1-integrin is required for maintaining the integrity of the glial tubes in the rostral migratory stream (Jacques et al., 1998; Belvindrah et al., 2007). SCO-spondin and SCO-spondin-derived compounds present in the CSF may reach the SVZ niche through the ependyma devoid of tight junctions. Due to its multidomain organization, SCO-spondin and its derivatives behave as a ligand for β1-integrin, collagen, and laminins of the ECM of the adult neurogenic niche. According to the evidence discussed above, these interactions could lead to changes in the microenvironment (basal lamina, ECM, growth factors, availability) and behavior of niche cells (NSC, neural progenitors, endothelial and ependymal cells; **Figure 6B**). Interestingly, bFGF, also secreted by SCO-cells, increases the expression of β1-integrin (Enenstein et al., 1992). Further, the effect of thyroid hormones on integrin signaling appears to be crucial for a normal neurogenesis (Stenzel et al., 2014). Cross-talking of SCO-spondin with other signaling pathways, such as those regulated by bFGF, thyroid hormones and low density lipoproteins could be envisaged.

## Experimental Approaches: Grafting of Subcommissural Organ to Promote Neurogenesis in the Adult SVZ Niche

**FIGURE 7:**
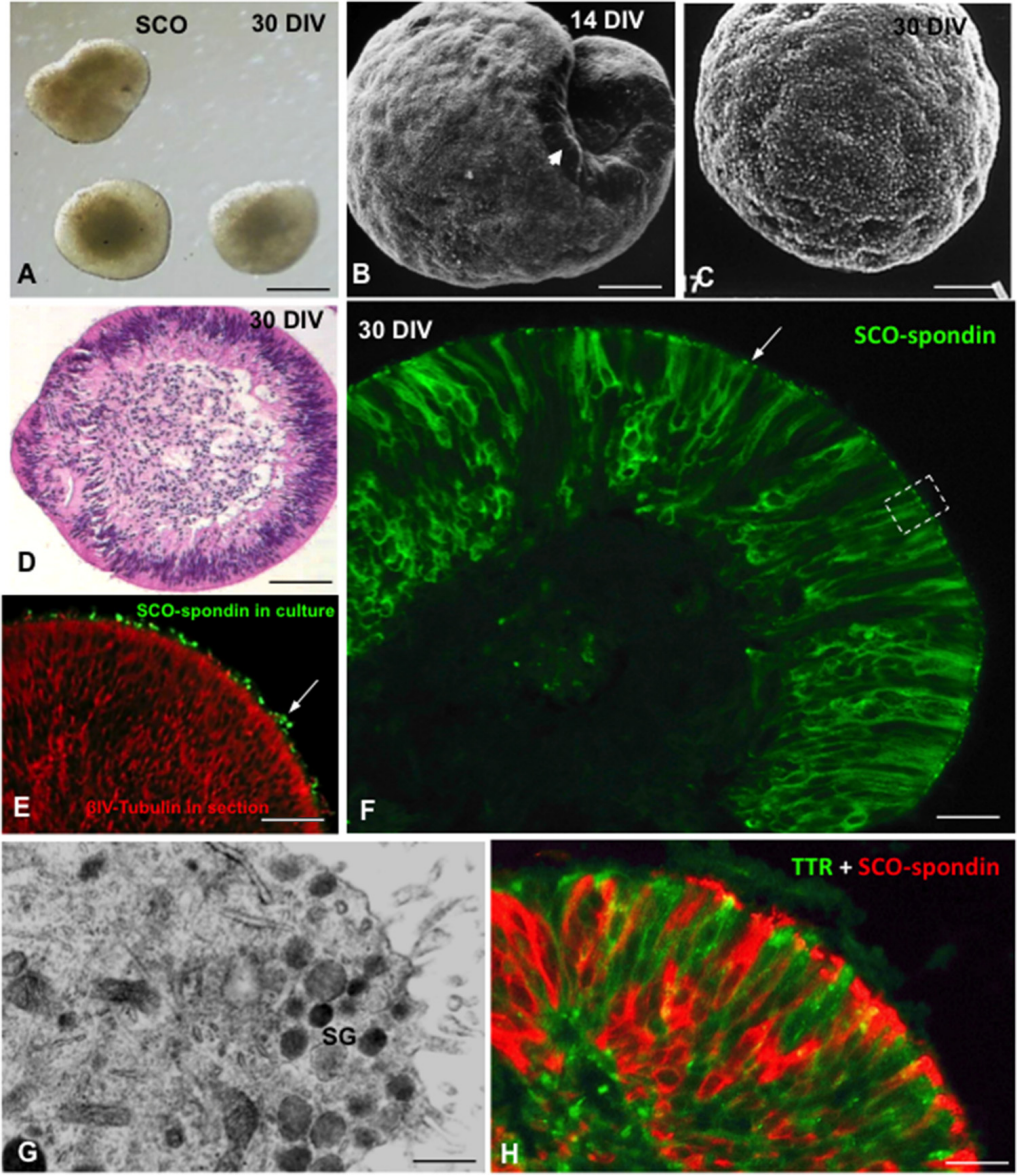
**Organ culture of bovine subcommissural organ.** After 30 days in culture, SCO explants organize forming spheres of secretory ependymocytes. **(A)** Phase contrast microscopy. **(B,C)** Scanning electron microscopy after 14 **(B)** and 30 DIV **(C)**. **(D)** Section of a SCO-explant stained with haematoxylin-eosin. **(E)** Secretory evidence of secretion. Explants were cultured in the presence of antibodies against SCO-spondin. After histological procedure, sections were incubated with anti-IgG conjugated with alexa 488. Immunofluorescence reveals the presence of SCO-spondin aggregates associated to cilia (green, arrow). **(F)** Section of a SCO-explant immunostained for SCO-spondin showing the intracellular and extracellular (arrow) location of the protein. **(G)** Ultrathin section of an area similar to that framed in previous figure, showing the ultrastructure of the apical cell pole loaded with secretory granules (sg). **(H)** Section of a SCO-explant. Double immunofluorescence for SCO-spondin (red) and TTR (green). Scale bars: **(A)** 60 μm; **(B–E)** 25 μm; **(F)** 10 μm; **(G)** 500 nm; **(H)** 10 μm. From Schöebitz et al. (2001), Montecinos et al. (2005).

Under proper culture conditions, SCO explants can be organ cultured for several months. After 3–4 weeks in culture, the explants form spheres lined by fully differentiated ependymal secretory cells (**Figure 7**). Explants synthetize (**Figure 7H**) and secrete SCO-spondin and TTR into the culture medium (Schöebitz et al., 2001; Montecinos et al., 2005).

Subcommissural organ explants grafted under the kidney capsule keep their secretory properties similar to the *in situ* SCO (Rodríguez et al., 1989). A network of processes of the perivascular basal lamina, resembling that found in circumventricular organs (Rodríguez, 1969; Rodríguez et al., 1979; Dellmann et al., 1987) and in the niche of the SVZ (Mercier et al., 2002; Kerever et al., 2007) connects the secretory cells to newly formed capillaries re-vascularizing the grafted SCO. Long-spacing collagen appears in expanded areas of such laminar networks and also in the perivascular space supporting that: (i) formation of long-spacing forms of collagen is triggered by factors provided by the SCO-secretory cells, and (ii) secretory material of the grafted ependymal and hypendymal cells reaches the extended network of the basal lamina processes (Rodríguez et al., 1989).

**FIGURE 8:**
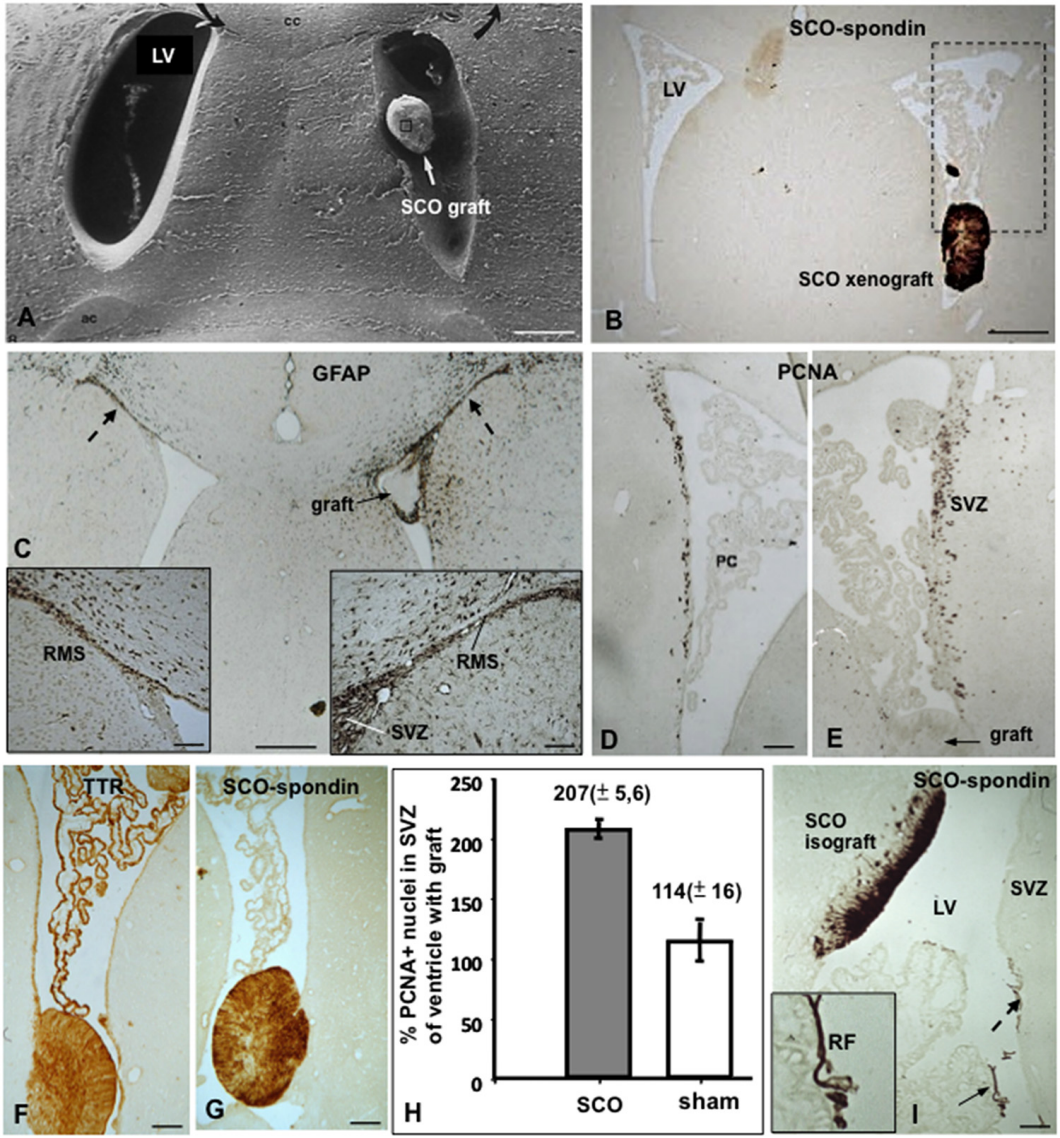
**Xeno- and isografting of SCO-explants into the lateral ventricle of adult rats. (A–H)** Bovine SCO-explants 30 DIV were grafted into the lateral ventricle of adult rats. **(A)** Scanning electron microscopy showing a SCO-explant in the ventricle. **(B)** Frontal section of the brain of a grafted animal immunostained with AFRU. The grafted SCO is strongly reactive. The area framed is shown in figures D and E. LV, lateral ventricle. **(C)** Frontal section of the brain of a grafted animal immunostained for GFAP. Astrocytes forming the rostral migratory stream (RMS) are shown. In the grafted ventricle the RMS is hypertrophied (right inset) as compared to that of the contralateral ventricle (left inside). **(D,E)** Areas similar to that framed in figure **(B)**, immunostained for PCNA. In the grafted ventricle **(E)** proliferation is significantly higher than in the contralateral ventricle **(D)**. **(F,G)** The grafted SCO expresses TTR and SCO-spondin. TTR is also expressed by the choroid plexus. **(H)** Quantitative analysis of PCNA+ nuclei after SCO grafting in a lateral ventricle of an adult normal rat. The results are expressed as percentage of the number of labeled nuclei in the SVZ of the ventricle carrying the grafts with respect to that of the contralateral ventricle, taken as 100%. Sham operated rats underwent surgery as for transplantation, but received no graft. There is a twofold increase of PCNA+ nuclei in the grafted ventricle. **(I)** Rat SCO explant grafted into the lateral ventricle of an adult rat. The graft becomes integrated into the wall of the lateral ventricle (LV) with the ependymal cells secreting SCO-spondin into the ventricle aggregated on the ependyma of the subventricular zone (broken arrow; SVZ) and forming a Reissner fiber (RF; full arrow; inset). Scale bars: **(A–C)** 120 μm; **(D,E)** 60 μm; **(F,G)** 40 μm; **(I)** 60 μm. From Rodríguez et al. (1999); González (2007).

Rat SCO explants grafted into a lateral ventricle of normal adult rats become re-vascularized and secrete RF-glycoproteins into the CSF forming a RF, now located in the lateral ventricle (**Figures 8A,B,F**). The basal lamina of the newly formed capillaries, but not the capillaries of the neighboring brain parenchyma, contains long spacing collagen, indicating that the expression of this special type of collagen is triggered by signals of the grafted SCO cells (Rodríguez et al., 1999). Xenografts of bovine SCO explants into a lateral ventricle of normal and hydrocephalic rats survive for weeks, secrete SCO-spondin and TTR to the host CSF and promote neurogenesis in the ipsilateral SVZ niche (Rodríguez et al., 1999; González, 2007; Jara et al., 2014; **Figures 8A–E,G–I**).

## Conclusion and Future Directions

A good body of evidence is revealing that the dynamic and molecular composition of the CSF and, consequently, the CSF physiology is much more complex and fascinating than the simplistic view held for decades. Signal molecules either specifically transported from blood to CSF or secreted into the CSF by a series of periventricular structures, use the CSF to reach their targets in the brain. This allows a cross talk between brain regions located beyond the blood-brain-barrier, thus keeping the brain milieu private. One of these brain target is the neurogenic niche, and the SCO, choroid plexus, and tanycytes are some of the sources of signals that reach this target via the CSF. Thus, the CSF path has made it possible for these four brain structures to become good functional partners.

As we unfold the multiples trans-fluid talks between discrete brain domains we will have more tools to influence, in one way or another, such talks. The CSF may become an appropriate medium to deliver foreign molecules or to host cell grafts.

## Conflict of Interest Statement

The authors declare that the research was conducted in the absence of any commercial or financial relationships that could be construed as a potential conflict of interest.

## Abbreviations

CSF: cerebrospinal fluid
ECM: extracellular matrix
FGF: fibroblast growth factor
NSCs: neural stem cells
RF: Reissner fiber
SCO: subcommissural organ
SVZ: subventricular zone
TTR: transthyretin
VZ: ventricular zone
